# Bitter Taste Receptors Expression in Human Granulosa and Cumulus Cells: New Perspectives in Female Fertility

**DOI:** 10.3390/cells10113127

**Published:** 2021-11-11

**Authors:** Bianca Semplici, Francesca Paola Luongo, Sofia Passaponti, Claudia Landi, Laura Governini, Giuseppe Morgante, Vincenzo De Leo, Paola Piomboni, Alice Luddi

**Affiliations:** 1 Department of Molecular and Developmental Medicine, University of Siena, 53100 Siena, Italy; semplici4@student.unisi.it (B.S.); francescapaola.lu@student.unisi.it (F.P.L.); giuseppe.morgante@unisi.it (G.M.); vincenzo.deleo@unisi.it (V.D.L.); paola.piomboni@unisi.it (P.P.); luddi@unisi.it (A.L.); 2 Department of Life Sciences, University of Siena, 53100 Siena, Italy; sofia.passaponti@student.unisi.it (S.P.); landi35@unisi.it (C.L.)

**Keywords:** bitter taste receptors, cumulus cells, granulosa cells, female fertility, oocyte quality

## Abstract

Bitter taste receptors (TAS2RS) expression is not restricted to the oral cavity and the presence of these receptors in the male reproductive system and sperm provides insights into their possible role in human reproduction. To elucidate the potential role of TAS2Rs in the female reproductive system, we investigated the expression and localization of bitter taste receptors and the components of signal transduction cascade involved in the pathway of taste receptors in somatic follicular cells obtained from women undergoing assisted reproductive techniques. We found that TAS2R genes are expressed in both cumulus (CCs) and granulosa (GCs) cells, with TAS2R14 being the most highly expressed bitter receptor subtype. Interestingly, a slight increase in the expression of TAS2R14 and TAS2R43 was shown in both GCs and CCs in young women (*p* < 0.05), while a negative correlation may be established between the number of oocytes collected at the pickup and the expression of TAS2R43. Regarding α-gustducin and α-transducin, two Gα subunits expressed in the taste buds on the tongue, we provide evidence for their expression in CCs and GCs, with α-gustducin showing two additional isoforms in GCs. Finally, we shed light on the possible downstream transduction pathway initiated by taste receptor activation in the female reproductive system. Our study, showing for the first time the expression of taste receptors in the somatic ovarian follicle cells, significantly extends the current knowledge of the biological role of TAS2Rs for human female fertility.

## 1. Introduction

Female fertility is the result of a finely controlled and specialized process during which the oocyte grows within the ovarian follicle, the functional unit that provides and influences the maturation and quality of the oocyte within it [1].

The preovulatory ovarian follicle is a follicular niche composed of highly differentiated cells (granulosa and cumulus cells) lining the antrum filled with follicular fluid. This complex biological fluid is derived from the ultrafiltration of serum and secretion of the cells lining the follicle itself (both granulosa and cumulus cells); the follicular fluid provides a very important microenvironment and contains regulatory molecules, such as protein hormones, steroids, and electrolytes, which are important for oocyte maturation and quality [2,3,4]. Oocyte competence, defined as the ability of the mature oocyte to withstand the development stage, from fertilization to embryo implantation in uterus, is therefore endured by the proper communication between the oocyte and surrounding somatic cells, granulosa, and cumulus cells [5]. The close connection between the somatic cells and the oocyte itself, which are structurally and metabolically dependent on each other, suggests that the health status of these cells is closely related to the quality of the oocyte [6,7,8]. In vitro studies have shown that granulosa–oocyte communication is essential for the normal oocyte growth; in fact, immature oocytes separated from granulosa cells do not grow [9].

The creation and maintenance of this specific microenvironment is also crucial for the fertilization since, at ovulation, the cumulus oocyte complex is released from the ovarian follicle together with part of the follicular fluid. Cumulus cells and follicular fluid can both actively participate in the fertilization process, by promoting or limiting the interaction between the cumulus oophorus and sperm at the level of the tubal ampulla [10]. In particular, these cells can release or recognize molecules acting as key biosensors in the chemosensation and/or guidance of sperm, such as progesterone [11]. Therefore, the ability to discriminate between all the molecules present in this microenvironment could be an advantage in terms of reproductive success.

To this regard, an intriguing perspective is represented by taste receptors (TASRs), transmembrane receptors capable of detecting umami, sweet, and bitter taste stimuli. Two different families of taste G protein coupled receptors (GPCRs) have been identified: Type 1 Taste Receptors (TAS1Rs) and Type 2 Taste Receptors (TAS2Rs) [12,13]. While TAS1Rs are responsible for the perception of sweet compounds and umami [14], TAS2Rs are responsible for the sensation of bitter tastants [15,16,17]. TAS2Rs are a large family including about 25 different isoforms in humans [18,19]. Taste receptors were first found in the taste buds of the oral cavity [20]; however, the extra-oral expression of taste receptors has been described in several recent reports [21,22,23]. Recently, the role of TAS2Rs receptors has been considered as a possible player for 2019-nCoV host defence mechanism. It was reported that TAS2R10 is involved in the control of infectious diseases caused by bacteria, viruses, and parasites, suggesting that TAS2R10 is a key trigger of host defence pathways [24].

Of note, their expression has also been reported in the male reproductive system [25,26,27], providing insight into their involvement in human fertility.

Our group demonstrated in a recent report the expression of TAS2Rs family members in ejaculated human sperm as well as in testicular tissue [28]. In addition, this report also provided evidence that molecules involved in taste signal transduction cascade, including the G protein α-subtypes gustducin and transducin and the enzymes PDE4A, PLCβ2, and TRPM5, are detected in the testicular tissue and ejaculated sperm [28]. The association of taste receptors gene expression with male infertility is realistic, because, to reach and fertilize the mature oocyte, mammalian sperm must undertake a long journey through the female genital tract. During this journey, mammalian sperm are exposed to a wide range of compounds of different origins and chemical properties [29,30,31,32].

To the best of our knowledge, no study has ever addressed the presence and expression of TAS2Rs in the human female reproductive system. To determine the possible involvement of taste receptors in female fertility, we have investigated, in both human granulosa and cumulus cells, the expression of five TAS2Rs, specifically TAS2R3, TAS2R4, TAS2R14, TAS2R19, and TAS2R43). Among the 25 taste receptors identified so far, we focused on these 5 because of their expression in the testis and in male gametes, as we already reported [28]. We also performed an in silico MetaCore pathway analysis, to depict a critical functional overview of TAS2Rs activities, thus outlining a complex and integrated functional protein-framework. The expression of molecules involved in signal transduction processes elicited by the activation of this class of receptors was also studied.

## 2. Materials and Methods

### 2.1. Samples Collection

For this study, we collected granulosa and cumulus cells from 25 women, diagnosed with tubal occlusion, who underwent in vitro fertilization at the UOSA of Assisted Reproductive Technique, Siena University Hospital. All enrolled patients were unable to conceive naturally for at least 1 year before entering the study. For data analysis, patients were randomly allocated to cohorts based on age (young ≤ 33, *n* = 15 and old ≥ 36, *n* = 10) and number of retrieved oocytes (≤5, *n* = 10 and ≥6, *n* = 15).

Ethical approval for the studies was obtained from the Siena University Hospital Local Ethical Committee (code 20170619, approval date 10 June 2017). All patients provided their informed consent before being enrolled in the study.

### 2.2. Ovulation Induction

Ovarian hyperstimulation was induced by subcutaneous administration of recombinant FSH from day 2 or 3 of the menstrual cycle, at a dose of 150–300 IU per day. The dose of gonadotropins was adjusted according to ovarian response, as detected by ultrasound examination and oestrogen serum level. A gonadotropin-releasing hormone (GnRH) antagonist was administered daily when the dominant follicle reached 14 mm in diameter. The ovulation triggering, planned when at least one follicle reached 18 mm in diameter, was obtained by administering recombinant human chorionic gonadotropin (hCG). The oocyte pickup was performed 34–36 h after the HCG injection. Follicular fluid was aspirated for cumulus oocyte complexes (COCs) and granulosa cells (GCs) recovery.

### 2.3. Granulosa and Cumulus Cells Isolation

After COCs isolation, follicular fluid samples were immediately processed to collect granulosa cells according to a previously described procedure [6]. Isolated cells were resuspended in Dulbecco Modified Eagle Medium (Invitrogen, Whaltman, MA, USA) supplemented with 10% fetal bovine serum, 2 mmol/L L-glutamine, 100 U/mL penicillin, and 100 μg/mL streptomycin, plated in a tissue culture dish and allowed to adhere for 24 h. Then, cells were collected and stored at −80 °C for following analyses. *CSF* gene expression was performed in the purified cells to confirm enrichment in GCs and removal of contaminating leukocytes. After oocyte retrieval, COCs were incubated in Continuous Single Culture Complete (CSCC) medium (Irvine Scientific, Inc. FujiFilm, Santa Ana, CA, USA) for ~2 h and then exposed to Hyaluronidase 80 IU (Irvine, Irvine Scientific, Inc. FujiFilm, Santa Ana, CA, USA) for 20 s. Cumulus cells (CCs) were stripped from the oocyte with the use of micropipettes (170 μm and 140 μm in diameter). The CCs from MII oocytes were immediately collected into pooled samples from each patient, washed two times, pelleted, and stored at −80 °C or fixed until following analyses.

### 2.4. RNA Isolation and Droplets Digital PCR Assay

Total RNA was isolated from both granulosa and cumulus cells by automatic extraction with RNeasy Protect Mini kit, according to the manufacturer’s instructions (Qiagen, Hilden, Germany). RNA quantity was assessed using an ND-1000 Nanodrop Spectrometer (Thermo Fisher Scientific, Wilmington, DE, USA); 100 ng of extracted RNA were reverse transcribed into cDNA using the iScript gDNA Clear cDNA Synthesis Kit (BioRad, Milan, Italy). Gene expression was evaluated using specific EvaGreen assays (Table S1), using the Bio-Rad’s QX200 ddPCR System (Bio-Rad, Hercules, CA, USA).

Droplets digital polymerase chain reaction (ddPCR) was performed in a total volume of 22 μL, containing 11 μL 2× QX200 ddPCR EvaGreen Supermix (Bio-Rad, Hercules, CA, USA), 1.1 μL PrimePCR EvaGreen Assays (Table S1), 8 μL RNase-free sterile water, and 2.5 μL diluted 1:10 cDNA. The mixture was added to the DG8 cartridge, followed by the loading of 70 μL of droplet generation oil for EvaGreen (Bio-Rad, Hercules, CA, USA), using an automated Droplet Generator (Bio-Rad, Hercules, CA, USA). Then, 40 µL of generated droplets were transferred into a 96-well PCR plate and heat-sealed with pierceable foil by using a PX1 PCR Plate Sealer (Bio-Rad, Hercules, CA, USA), for 3 s at 175 °C twice before thermal cycling, and then placed in a thermal cycler (T100 Thermal Cycler, Bio-Rad, Hercules, CA, USA). The cycling conditions were as follows: 5 min at 95 °C, 30 s at 96 °C, 1 min at 58 °C, 5 min at 4 °C, and 5 min at 90 °C. After PCR, the 96-well PCR plate was loaded into the QX200 Droplet Reader (Bio-Rad, Hercules, CA, USA), to identify the fluorescence intensity of each droplet for EvaGreen fluorophore. For each RNA specimen, a negative control was prepared by omitting the reverse transcriptase. Data were analyzed using the QuantaSoftTM Analysis Pro software, version 1.0 (Bio-Rad, Hercules, CA, USA). A threshold line was employed to discriminate positive and negative droplets. The Poisson statistics were applied to calculate the absolute concentration of each target gene in copies/μL. Three reference genes (Table S1) were used to normalize the RNA amount, obtaining a final value of relative gene expression, expressed as normalized sample amount (NSA).

### 2.5. Western Blot Analysis

For Western blotting, 50 μg of total proteins from each sample were diluted in Laemmli buffer (100 mM Tris-HCl pH 6.8, 2% *w*/*v* SDS, 20% *v*/*v* glycerol, 4% *v*/*v* β-mercaptoethanol), kept at 95 °C for 5 min and loaded and separated on 10% polyacrylamide gel using the Cell Mini Protean (BioRad Microsciences, Hemel Hempstead, UK). The gel was transferred onto nitrocellulose membrane Hybond ECL, (GE Healthcare, Chicago Illinois, IL, USA) in a mini Trans-Blot apparatus (Bio-Rad, Hercules, CA, USA). Membranes were subsequently blocked with 5% nonfat dry milk in TBS (Tris-buffered saline, 10 mM Tris-HCl, pH 7.5 and 0.15 M NaCl) for 1 h at room temperature, and then the incubation with primary antibodies (see Table S2) diluted in 1% nonfat dry milk/TTBS (TBS containing 0.2% Tween 20) was carried out overnight at 4 °C. After washing, membranes were incubated for 1 h with the appropriate horseradish peroxidase (HRP)-conjugated secondary antibody (see Table S2). As an internal loading control, the same nitrocellulose membranes were also incubated with an anti-actin antibody, followed by the appropriate secondary antibody. Immunostained bands were visualized by chemiluminescence with Image Quant LAS 4000 (GE Healthcare, Chicago, Illinois, IL, USA). Band density was quantified with ChemiDoc (Bio-Rad Microsciences, Hemel Hempstead, UK).

### 2.6. Immunofluorescence

GCs and CCs were cultivated on glass coverslips, washed with PBS, fixed for 10 min in 4% paraformaldehyde (PFA; Sigma-Aldrich, Milano, Italia) at RT and then washed 3 times with PBS. Subsequently, the samples on coverslips were permeabilized in 0.5% Triton X-100 in PBS for 10 min, washed three times with PBS, and immunolabeled using an indirect procedure. All incubations were performed in blocking solution containing 5% Bovine Serum Albumin (BSA). Specificity of immunostaining was confirmed by both omission of primary antibody and staining of sections with unrelated antibodies (Figure S1). Coverslips were incubated for 1 h at RT with the primary antibody (Table S2) and then washed three times in PBS; the primary antibody binding was visualized by incubation with a secondary antibody (Table S2). After washing three times with PBS, the cell nuclei were stained with 4′,6-Diamidine-2′-phenylindole dihydrochloride (DAPI) for 15 min at RT; finally, the coverslips were mounted with antifade solution and observed with a Leica DMB 6000 microscope. Images were captured with a CFTR6500 digital camera (Leica, Microsystem, Germany).

### 2.7. MetaCore Analysis

The gene names of selected proteins were submitted to the MetaCore network building tool (Thomson Reuters, New York City, NY, USA) software to find their functional activity and the functional correlation existing among them. MetaCore included a manually annotated database of protein interactions and metabolic reactions obtained by scientific literature. Gene names of all identified proteins were imported into MetaCore and processed using the shortest path algorithm; consequently, only those proteins known to be closely related were included in the resulting path. Hypothetical networks were built among the experimental proteins and the MetaCore proteins database. The relevant pathway maps were then prioritized according to their statistical significance (*p* ≤ 0.001) and networks were graphically visualized as nodes (proteins) and edges (the relationship between proteins). This analysis suggested the biochemical context in which the proteins of interest act, and how their aberrant expression may alter cellular and/or tissue biology in the disease status.

### 2.8. Statistical Analysis

Statistical analysis was performed using the GraphPad Prism 5.0 (GraphPad Software, San Diego, CA, USA). Statistical significance was evaluated by using nonparametric tests. Differences among groups of data were tested by Mann–Whitney test for two groups or Kruskal–Wallis one-way analysis of variance followed by the Dunn’s post-hoc test for multiple comparisons. Correlation was determined by using Spearman’s test. Statistical significance was set at *p* < 0.05.

## 3. Results

### 3.1. Gene Expression Analysis of TAS2Rs in Granulosa and Cumulus Cells

Cumulus and granulosa cells were isolated from the cumulus–oocyte complex and follicular fluid of patients undergoing assisted reproduction, in whom the recovered oocyte pool included only mature metaphase II stage.

To clarify the role of TAS2Rs in the somatic cells of the ovarian follicle, mRNA levels were measured in both CCs and GCs. The normalized sample amount (NSA) of each gene was obtained by using *TBP*, *HPRT1*, and *PPIB* as reference genes [6].

**Figure 1:**
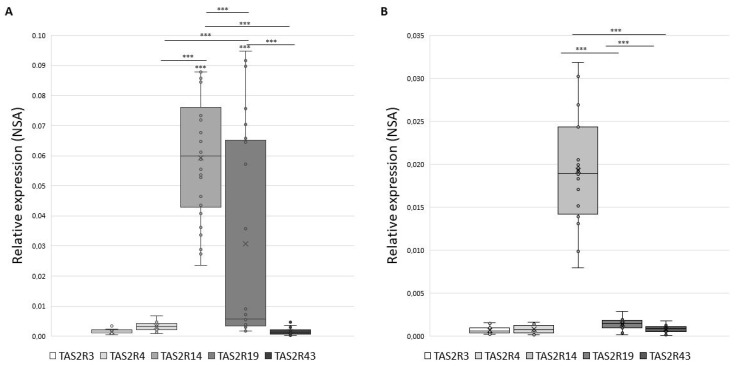
Gene expression of *TAS2Rs* in GCs panel (**A**) and in CCs panel (**B**) expressed as Normalize Sample Amount. Graphical diagrams are plotted as box–whisker plots, where boxes show the interquartile range with median and mean values, and whiskers represent min and max confidence intervals. Statistically significant differences in NSA levels were tested by Kruskal–Wallis one-way analysis of variance followed by the Dunn’s post-hoc test. *** *p* < 0.001.

Figure 1 shows the distribution of NSA values in GCs (panel A) and in CCs (panel B) that appears to be significantly modulated, with *TAS2R14* being the most expressed in both GCs (mean NSA = 0.0595) and in CCs (mean NSA = 0.0197) (*p* < 0.001).

An inter-individual variability for single *TAS2R* must be disclosed: while we detected the expression of *TAS2R4* and *TAS2R14* in 100% of samples, *TAS2R19* and *TAS2R43* were detected in about 96% of samples, and *TAS2R3* in 85% of GCs samples. Analogously, in CCs only *TAS2R14* was expressed in 100% of tested samples, while *TAS2R4*, *TAS2R19*, and *TAS2R43* were expressed in about 89% and *TAS2R3* in 79% of tested CCs samples. Therefore, TAS2R3 exhibits the highest inter-individual variability in both GCs and CCs.

**Figure 2:**
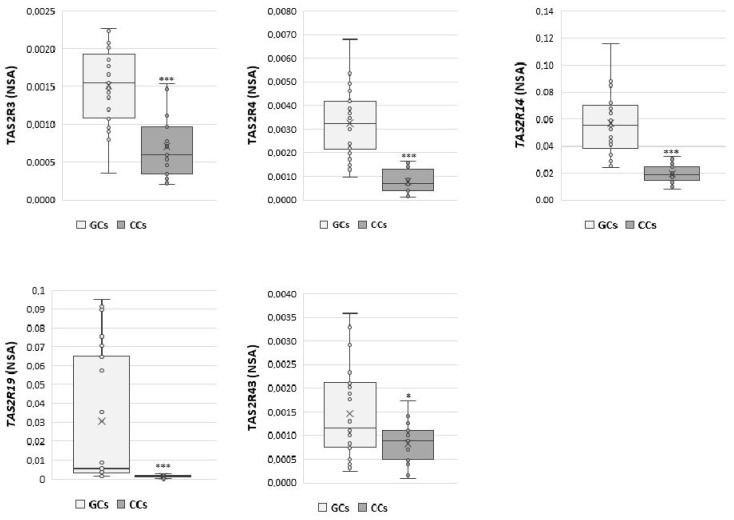
NSA levels in GCs and CCs for *TAS2R3*, *TAS2R4*, *TAS2R14*, *TAS2R19,* and *TAS2R43*. Graphical diagrams are plotted as box–whisker plots, where boxes show the interquartile range with median and mean values, and whiskers represent min and max confidence intervals. Statistically significant differences in mRNA levels were tested by Mann–Whitney test. * *p* < 0.05, *** *p* < 0.001.

Moreover, direct comparison between the expression of each gene in GCs and CCs (Figure 2) confirms the different expression levels of *TAS2Rs* genes in GCs and CCs that may be dependent on the specific cell differentiation.

The expression of *TAS2R3* was significantly higher in GCs than in CCs (*p* < 0.001). Additionally, *TAS2R4* and *TAS2R14* were significantly up regulated in GCs compared with CCs (*p* < 0.001), and the same behaviour was observed for *TAS2R19*, which appeared nearly undetectable in cumulus cells (*p* < 0.001). Finally, we found a slightly significant difference in *TAS2R43* expression between GCs and CCs (*p* ≤ 0.05).

**Figure 3:**
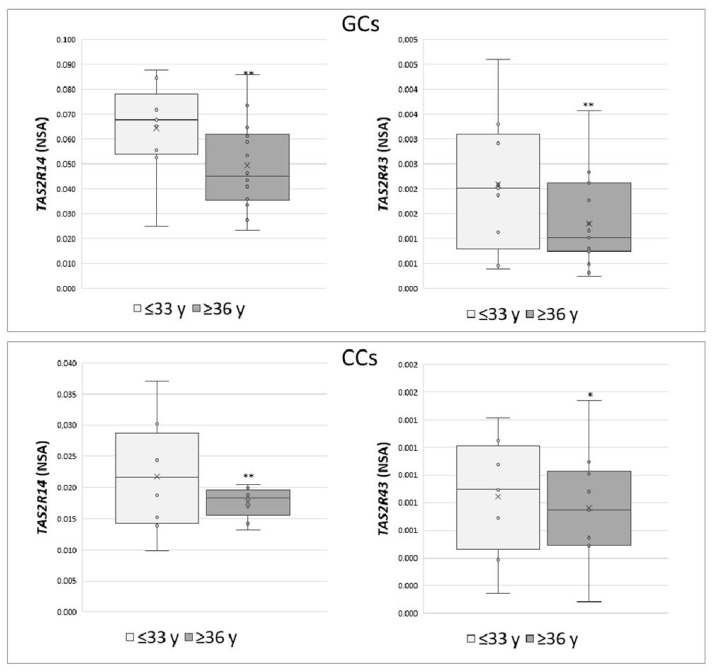
Relative NSA levels in GCs and CCs for *TAS2R14* and *TAS2R43*. Graphical diagrams are plotted as box–whisker plots, where boxes show the interquartile range with median and mean values, and whiskers represent min and max confidence intervals. Statistically significant differences in mRNA levels were tested by Mann–Whitney test. * *p* < 0.05, ** *p* < 0.01.

These data were also analysed according to specific parameters potentially affecting IVF outcome, such as patient age (young women ≤33 years vs. old women ≥36 years) and number of retrieved oocytes (poor responders ≤5 vs. high responders ≥6). An increase in the expression of *TAS2R14* and *TAS2R43* was highlighted in both GCs and CCs in young women (*p* < 0.05) (Figure 3), while a negative correlation was found between the number of collected oocytes and the NSA level of *TAS2R43* in cumulus cells (r = −0.6, *p* < 0.01).

### 3.2. Protein Quantification and Localization of TAS2Rs

**Figure 4:**
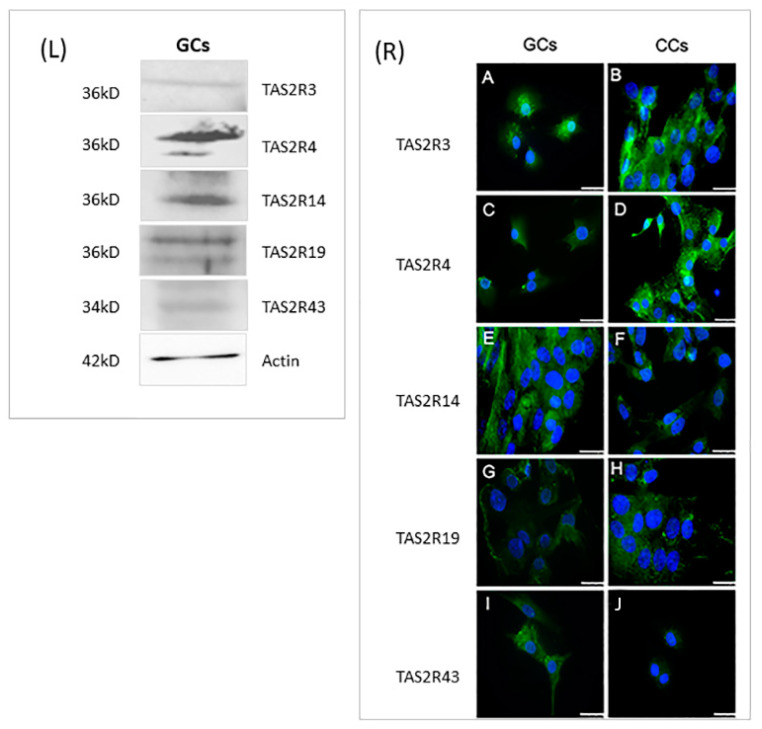
Left panel (**L**): Representative image of Western blot of TAS2R3, TAS2R4, TAS2R14, TAS2R19, and TAS2R43. β-actin was used as loading control. Right panel (**R**): Immunofluorescence localization of (**A**,**B**) TAS2R3, (**C**,**D**) TAS2R4, (**E**,**F**) TAS2R14, (**G**,**H**) TASR19, and (**I**,**J**) TAS2R43 in (**A**,**C**,**E**,**G**,**I**) granulosa and (**B**,**D**,**F**,**H**,**J**) cumulus cells. TAS2Rs are stained in green. Nuclei were counterstained with DAPI (blue). Scale bar = 15 µm. Negative controls are shown in Figure S1.

In order to confirm, at the protein level, the presence of selected TAS2Rs, we analyzed by Western blotting protein extracts prepared from GCs, obtained by pooling these cells from the same patients we used for gene expression analysis. Unfortunately, we were not able to perform Western blot analysis in CCs because of the low protein amounts that we collected from each patient and, at the same time, because of the low gene expression level of these receptors in CCs. The results confirmed the data obtained in GCs by gene expression analysis; indeed, all proteins were detectable in GCs extracts. Although we cannot assure that the detection efficiencies of the different antisera that we used are equivalent, we may observe that both TAS2R3 and TAS2R43 are the least expressed receptors (Figure 4L). These data are in line with previous gene expression analysis (Figure 1)

Cellular localization of selected TAS2Rs was evaluated in both CCs and GCs, through immunofluorescence (Figure 4R). The localization of TAS2R3 showed in CCs a signal distributed throughout the cytoplasm (Figure 4R(A)), while in GCs, it is also localized around the nucleus (Figure 4R(B)). TAS2R4 protein showed an intense and dispersed signal with some sparse fluorescent granules in CCs (Figure 4R(C)), while in GCs, a less intense signal was present (Figure 4R(D)). Regarding TAS2R14, immunofluorescence staining was concentrated around the nuclear envelope in both GCs and CCs (Figure 4R(E,F)). The analysis of TAS2R19 showed a diffuse and intense signal around the nuclei in both cell types, with the presence of some fluorescent granules, suggesting an accumulation of this protein inside vesicles (Figure 4R(G,H)). Immunostaining of TAS2R43, points out a weak staining in the cytoplasm. There is a great accumulation of fluorescent granules, especially in the cortical region of GCs, therefore suggesting a possible involvement of the protein in membrane trafficking (Figure 4R(I,L)).

### 3.3. MetaCore Protein Network Analysis of Protein Involved in TAS2Rs Signaling

**Figure 5:**
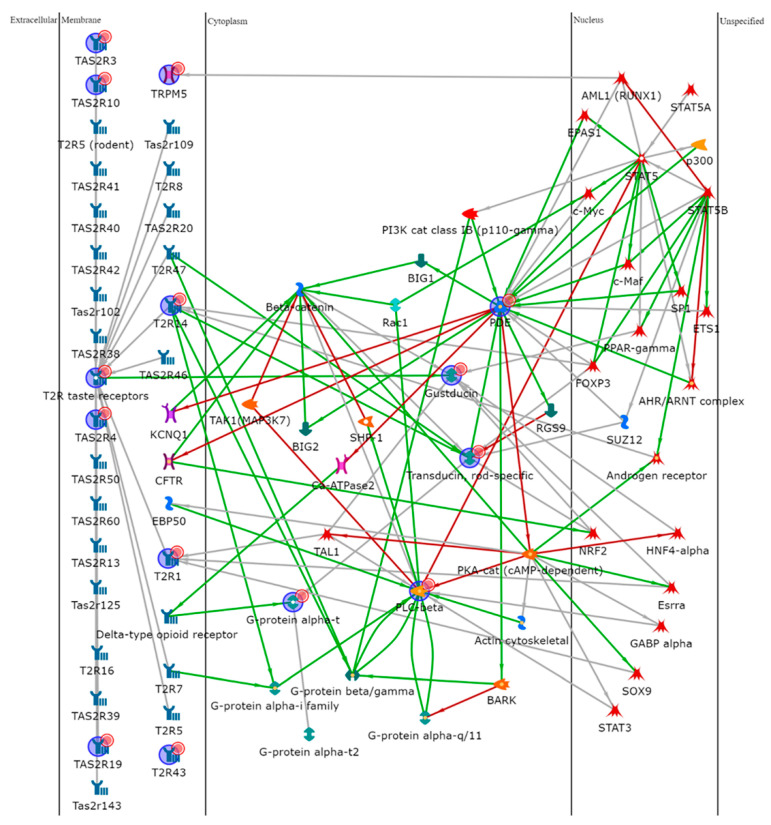
Protein network analysis of all the factors involved in this study, made using MetaCore software. PDE4A, PLCB2, GNAT1, GNAT3, and T2R14 were central functional hubs of the net built by shortest path algorithm. Only closely related proteins were included in the resulting path prioritized according to their statistical significance (*p* ≤ 0.001).

To further explore the role of the TAS2Rs, we performed an enrichment analysis uploading the gene names of the different isoforms on MetaCore software. Data were elaborated by shortest-path algorithm to build a theoretical biological network in which proteins potentially involved in the taste signal transduction were represented. Figure 5 shows the resulting pathway in which proteins significantly related to the taste receptors are reported. Software processing developed a network with α-Gustducin (GNAT3), α-Transducin (GNAT1), phosphodiesterase (PDE), and Phospholipase C beta (PLC beta) as “central hubs” (i.e., proteins interacting with five or more edges of the network). Transcription factors, in particular Signal Transducer and Activator of Transcription (STAT), c-myc, Peroxisome proliferator-activated receptor gamma (PPAR-γ), and Androgen receptor (AR) were involved in the network by potential interactions with many of the identified proteins.

### 3.4. Expression Profile of Genes Involved in the Signal Transduction Elicited by TAS2Rs

Based on the data obtained by the MetaCore analysis as well as on the data from the literature, we focused our attention on the expression of several genes involved in the signal transduction cascade elicited by TAS receptors: membrane and cytoplasmic associated factors (*GNAT1*, *GNAT3*, *PDE4A*, *TRPM5*, *PLCB2*) as well as nuclear factors (*STAT5a*, *STAT5b*, *PPARgamma*, *c-myc*, *GABPa*, and *AR*).

**Figure 6:**
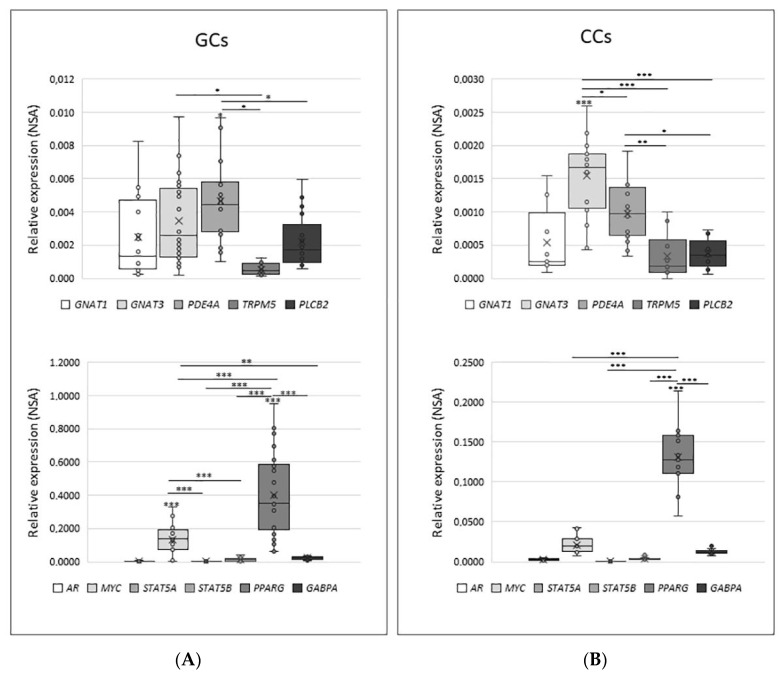
Relative quantity expression of genes involved in transduction cascade in GCs panel (**A**) and in CCs panel (**B**). Graphical diagrams are plotted as box–whisker plots, where boxes show the interquartile range with median and mean values, and whiskers represent min and max confidence intervals. Statistically significant differences in NSA levels were tested by Kruskal–Wallis one-way analysis of variance followed by the Dunn’s post-hoc test. * *p* < 0.05, ** *p* < 0.01, *** *p* < 0.001.

As shown in Figure 6, the distribution of NSA values in both GCs and CCs has a significant variation; indeed, between cytoplasmic factors, *PDE4A* is the highest expressed gene in GCs (mean NSA = 0.00466), while *GNAT3* is the highest expressed gene in CCs (mean NSA = 0.00154). Regarding nuclear factors, they appear to be significantly modulated, with *PPARG* being the most expressed in both GCs (mean = 0.4023) and in CCs (mean = 0.1306).

The direct comparison between the expression of each gene involved in the signal transduction cascade in GCs and CCs confirms the trend already reported for the expression of taste receptors in these cells. Indeed, all investigated genes have significantly higher expression in GCs than in CCs (*p*< 0.05 or *p* < 0.001), except for AR and STAT5a, which are expressed at the same level in both cell types. When the expression level was analysed according to specific parameters potentially affecting IVF outcome, such as patient age (young ≤33 vs. old ≥36) and number of retrieved oocytes (poor responders, ≤5 vs. high responder, ≥6), we only found a slightly significant increase for *AR* in GCs and for *PLCB2* and *MYC* in CCs (*p* < 0.05) from older women (data not shown).

To evaluate any possible correlation of *TAS2Rs* expression with the genes involved in their signalling pathway, the mean of *TAS2Rs* was calculated for each sample, and then analysed in both GCs and CCs. Regarding GCs, it was observed a positive correlation between *TAS2Rs* expression and *GNAT3* expression (r = 0.68; *p* < 0.001). Each *TAS2Rs* single subset was then correlated with the genes involved in the signalling pathway, and interestingly, *TAS2R14* was found to be the most correlated with *GNAT3* (r = 0.30; *p* < 0.001). This could be related to the high expression of *TAS2R14*.

### 3.5. Protein Quantification and Localization of Gustducin and Transducin

The presence of two of the most important G-proteins coupled to bitter taste receptors α-transducin and α-gustducin was confirmed at the protein level. The central hubs obligate mediators of all signal transduction elicited by TAS2Rs receptors; we analyzed by Western blotting protein extracts prepared from GCs and CCs, obtained by pooling these cells from the same patients we used for gene expression analysis. The results partially confirmed the data obtained by gene expression. 

**Figure 7:**
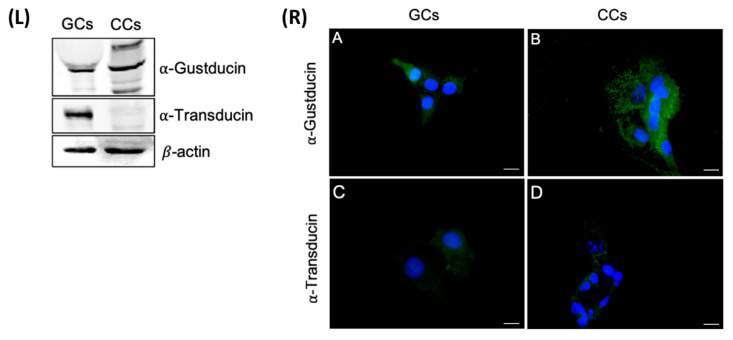
Left panel (**L**) The upper panel shows the detection of α-gustducin by Western blot with an anti-α-gustducin antibody, in CCs and GCs protein extracts. The central panel shows the detection of α-transducin by Western blot with an anti-α-transducin antibody in GCs and CCs protein extracts. β-actin was used as loading control. Right panel (**R**) Immunofluorescence localization of (**A**,**B**) α-gustducin, (**C**,**D**) α-transducin, in (**A**,**C**) granulosa and (**B**,**D**) cumulus cells. α-gustducin and α-transducin are stained in green. Nuclei were counterstained with DAPI (blue). Negative controls are shown in Figure S1. Scale bar = 15 µm.

In regard to α-gustducin, a single band of the predicted molecular weight of about 40 kDa was present in CCs, whereas in GCs, in addition to the presumed band corresponding to α-gustducin, we detected two other isoforms: one band at about 60 kDa, which recently has been described, in taste tissue preparations, to represent α-gustducin within insoluble complexes [33], and the other, less intense, at about 25 kDa. As regards α-transducin, a strong band of the expected molecular size (approximately 50 kDa) was present in CGCs, but not in CCs extracts (Figure 7, left panel).

The cellular localization of α-transducin and α-gustducin has been evaluated both in GCs and CCs. Immunofluorescence staining revealed a diffuse cytoplasmic signal for α-gustducin, confirming the Western blot analysis. α-gustducin showed a strong scattered signal at the cell periphery, consistent with a sub-membrane localization in CCs; in GCs, α-transducin revealed a very faint signal, as confirmed in Western blot analysis (Figure 7, right panel).

## 4. Discussion

This study, for the first time, demonstrates the expression of bitter TAS2Rs in human granulosa and cumulus cells. Furthermore, the expression of individual components of the taste signal transduction cascade, such as the α gustducin and transducin subtypes, and the effector enzymes and transcription factors, was observed in both cell types.

Expression of taste receptor genes has also been reported in non-taste tissues, suggesting that their role in the oral cavity does not represent the totality of their signaling capacity [34,35,36,37,38,39]. Indeed, it has become increasingly evident that the role of bitter taste perception may not be exclusively related to dietary habits, such as preventing the ingestion of toxic secondary metabolites [40]. In this regard, the already reported presence of these receptors in the male reproductive system [23,26,28,39] provides insights into their possible role in human reproduction. Furthermore, their differential expression in some diseases such as alteration of thyroid function [41], schizophrenia [42], and Parkinson disease [43] suggests the intriguing possibility that TAS2Rs could be used as valuable pharmacological targets in pathological conditions [23].

To the best of our knowledge, data regarding TASRs expression in the female reproductive system is lacking or conflicting. We provide for the first time evidence on the expression of TAS2Rs in GCs and CCs, the somatic cells surrounding the oocyte, which are considered critical for oocyte development and the fertilization process.

Interestingly, we observed a high inter-individual variability in the expression of TAS2Rs subtypes, with only TAS2R14 expressed in 100% of tested samples, in both CCs and GCs. These data, despite referring to only 5 of the 25 bitter receptors described in the literature, are in agreement with a study showing that each taste receptor cell expresses a different subset of receptor genes [44]. This implies that cells expressing one specific TAS2R member may have stronger/lower responses than cells carrying a different TAS2 receptor.

We also demonstrated that selected TAS2Rs are expressed at higher levels in GCs than in CCs, confirming that these cells, despite the same origin, are able to acquire a highly specialized profile. Of note, we highlighted *TAS2R14* as the most expressed gene in both GCs and CCs. This result allows to hypothesize a key role of this receptor in follicular cells’ physiology and, consequently, in human reproduction. This hypothesis is also supported by a study showing that *TAS2R14* is correlated with sperm progressive motility [26].

TAS2R14 is specifically activated by resveratrol [45,46,47], a natural polyphenol, with antidiabetic, antioxidant, and anti-inflammatory actions [48,49].

At the ovarian level, resveratrol supplementation has been reported to increase the total oocytes number, decrease atretic follicles, and decrease the apoptotic index of the granulosa cells in rats [50,51]. In addition, resveratrol protected oocytes by decreasing reactive oxygen species (ROS) production [52] Our data, highlighting TAS2R14 as the most highly expressed TAS2R gene in cumulus and granulosa cells suggests a possible mechanism by which resveratrol may exert potential beneficial actions on reproductive functions and ovaries.

Of note, *TAS2R14* is significantly upregulated in young compared with older women, again confirming its possible involvement in female fertility. However, conflicting data have been published demonstrating that noscapine (NOS), an agonist of the human TAS2R14, can induce apoptosis in human ovarian cancer cells not only through its documented alteration of microtubule assembly dynamics [53], but also through activation of TAS2R14 [40].

Although taste receptors are also reported to be expressed in non-taste tissues, functional analysis of TAS2Rs in extra-oral tissues remains a challenge. Our in silico analysis using Metacore software sheds light on the molecular pathway possibly activated by the binding between TAS2Rs and their ligands. Considering these observations, our analysis of the components of the signal transduction cascade in somatic cells of the ovarian follicle is very interesting. Our results showed a slightly higher expression of the investigated genes in GCs, confirming the trend shown for *TAS2Rs*. Our observations are in agreement with several studies showing that the transcriptome of GCs and CCs is distinctive for each cell population, giving a possible explanation for the different expression pattern observed [54,55].

Among the transcription factors identified by the Metacore analysis, PPARγ is the most highly expressed in both GCs and CCs, confirming literature data reporting this factor as being highly expressed in the granulosa cells, where it is primarily responsible for both estradiol production and regulation of follicular fluid content [56,57].

In this regard, a recent study has shown that TAS2Rs associate the detection of bitter-tasting molecules with changes in thyrocyte function and T3/T4 production [41]. This leads us to hypothesize and investigate the existence of a similar mechanism that may link bitter taste receptors to the synthesis of FSH and LH hormones, produced by granulosa and cumulus cells, respectively.

## 5. Conclusions

In light of these observations, our data pave the way for understanding the biological functions exerted by these receptors in the female reproductive tract. Future studies are needed to shed light on the molecular mechanisms triggered by these receptors in the follicular microenvironment. This knowledge could be useful to improve in vitro fertilization techniques currently in use, as well as potential therapeutic approaches concerning the treatment of human infertility.

## Data Availability

The raw data supporting the conclusions of this article will be made available by the authors without undue reservation.

## Supplementary Materials

The following are available online at https://www.mdpi.com/article/10.3390/cells10113127/s1, Figure S1: Representative images of negative controls for the immunofluorescent staining of GCs (A,C) and CCs (B,D) showed in Figure 4 and Figure 7. Bar = 25 µm; Table S1: PrimePCRddPCR Gene Expression Probe Assays, specific for ddPCR; Table S2: List of antibodies used in this study.
