# Supplementary material for: Bitter Taste Receptors Expression in Human Granulosa and Cumulus Cells: New Perspectives in Female Fertility
Source: Cells. 2021 Nov 11;10(11):3127. doi: 10.3390/cells10113127 (PMC8619861; doi:10.3390/cells10113127)
Supplement: Supplementary file 1 [file cells-10-03127-s001.zip › cells-1432272-supplementary.pdf]

Table S1. PrimePCRddPCR Gene Expression Probe Assays, specific for ddPCR.

Target Genes	Acronym	ID assay Probe
Taste receptor, type 2, member 3	*TAS2R3*	dHsaCPE5028212
Taste receptor, type 2, member 4	*TAS2R4*	dHsaCPE5028214
Taste receptor, type 2, member 14	*TAS2R14*	dHsaCPE5043852
Taste receptor, type 2, member 19	*TAS2R19*	dHsaCPE5027704
Taste receptor, type 2, member 43	*TAS2R43*	dHsaCPE5192389
G protein subunit alpha transducin 1	*GNAT1*	dHsaCPE5025595
G protein subunit alpha transducin 3	*GNAT3*	dHsaCPE5043953
Phosphodiesterase 4A, cAMP-specific	*PDE4A*	dHsaCPE5046851
Phospholipase C, beta 2	*PLCB2*	dHsaCPE5047389
Transient receptor potential channel, subfamily M, member 5	*TRPM5*	dHsaCPE5032805
Signal Transducer And Activator Of Transcription 5A	*STAT5A*	dHsaCPE5026004
Signal Transducer And Activator Of Transcription 5b	*STAT5B*	dRnoCPE5167406
Peroxisome proliferator-activated receptor gamma	*PPARγ*	dHsaCPE5036470
GA Binding Protein Transcription Factor Subunit Alpha	*GABPa*	dHsaEG5016715
Androgen Receptor	*AR*	dHsaCPE54526914
**Reference Genes**	**Acronym**	**ID assay Probe**
Hypoxanthine-Guanine Phosphoribosyl Transferase 1	*HPRT1*	dHsaCPE5192871
Tata Binding Protein	*TBP*	dHsaCPE5058363
Peptidylprolyl isomerase B (cyclophilin B)	*PPIB*	dHsaCPE5055049

Table S2. List of antibodies used in this study.

Antigen	Donor species	Dilution		Manufacturer	RRID
		IF	WB		
Primary antibodies					
**β-Actin**	Mouse	/	1:2000	Bio-Rad Laboratories	AB_2223350
α-Gustducin	Rabbit	1:100	1:500	Santa Cruz	AB_673678
α-Transducin	Rabbit	1:100	1:500	Santa Cruz	AB_2279097
TAS2R3	Rabbit	1:100	1:500	Thermo Fisher Scientific	AB_2556263
TAS2R4	Rabbit	1:100	1:500	Thermo Fisher Scientific	AB_2201090
TAS2R14	Rabbit	1:100	1:500	Novusbio Laboratories	AB_11053160
TAS2R19	Rabbit	1:300	1:1000	Thermo Fisher Scientific	AB_962285
TAS2R43	Rabbit	1:300	1:1000	Thermo Fisher Scientific	AB_2815963
Secondary antibodies					
Anti-Rabbit-IgG FITC	Goat	1:2000	-	Bio-Rad Laboratories	AB_1163662
Anti-Mouse-IgG HRP	Goat	-	1:8000	Bio-Rad Laboratories	AB_609692
Anti-Rabbit-IgG HRP	Goat	-	1:8000	Bio-Rad Laboratories	AB_1102634

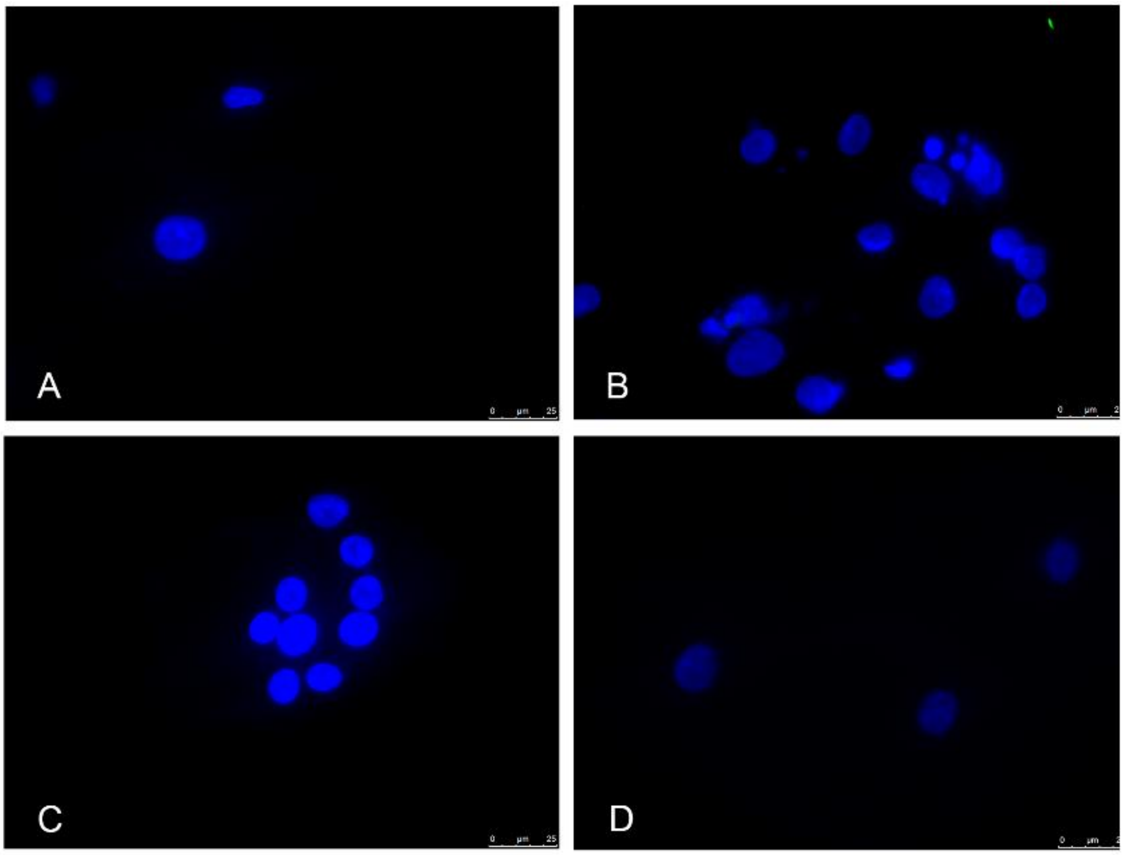

Figure S1. Representative images of negative controls for the immunofluorescent staining of GCs (A,C) and CCs (B,D) showed in Figure 4 and Figure 7. Bar = 25μm.
